# Humanism in the Age of COVID-19: Renewing Focus on Communication and Compassion

**DOI:** 10.5811/westjem.2020.4.47596

**Published:** 2020-04-24

**Authors:** Jonathan D. Sonis, Maura Kennedy, Emily L. Aaronson, Joshua J. Baugh, Ali S. Raja, Bryan J. Yun, Benjamin A. White

**Affiliations:** Massachusetts General Hospital, Harvard Medical School, Department of Emergency Medicine, Boston, Massachusetts

## INTRODUCTION/BACKGROUND

The global novel coronavirus (COVID-19) pandemic continues to worsen and has become one of the largest clinical and operational challenges faced by emergency medicine since its inception as a specialty. As the virus spreads across the United States, our emergency departments (ED) continue to see increased volumes of infected patients, many of whom are not only critically ill, but acutely aware and fearful of their circumstances and potential mortality. Given the significant fear, anxiety, and uncertainty that has accompanied this pandemic, there may be no more important time than now to focus on staff-patient communication and the expression of empathy and compassion.

Unfortunately, while patients and their families often face terrifying and isolating circumstances, many of the techniques ingrained in us as emergency clinicians to comfort patients in times of uncertainty are also challenged by the need for self-preservation from this disease. Infection precautions, while critical to reduce the spread of COVID-19, are by their very nature “isolating,” and make placing a hand on a patient’s shoulder, sitting at the foot of the bed, or spending a few extra minutes in a patient’s room much less likely to occur. In addition, many of the epidemiological techniques and interventions aimed at reducing the spread of disease (eg, social distancing, reduced in-person interactions) can mean both that the arriving patient already feels some degree of isolation and that the clinicians caring for them are primed to continue a more distanced approach.

Further, as we attempt to preserve personal protective equipment (PPE) in the face of a critical nationwide shortage, many EDs have moved to use telephone and video technology to interview, reassess, and educate our patients; and therefore, even the comfort provided by the doctor “walking in” may be lost. This may serve – explicitly or implicitly – to increase a patient’s sense of isolation and even stigmatization. And the interpersonal isolation experienced by our patients is even more pronounced outside the walls of the ED. For those well enough to return home and wait, hoping for improvement and not clinical decline, our instructions are clear: stay at home, alone, and isolate yourself from everyone – even your own family.

For all of these reasons, the burden of COVID-19 extends far beyond the physiologic manifestations. While the social and psychological scars that this pandemic will leave on all of us remain to be seen, the decreased compassion and humanism experienced by our patients is unquestionable. Yet, even while avoiding direct physical contact, wearing PPE, limiting in-person communication, and demanding social isolation, opportunity still exists to express the empathy that led us to the practice of medicine far before the age of COVID-19. But, while ED providers and staff are stretched so thin – both professionally and emotionally – is it practical to put another “ask” on our plates?

We argue that emphasizing compassion and humanism in our current circumstances will not be burdensome for our staff, but rather improve our own job and personal satisfaction during this challenging time. As Dr. Thomas Lee, an internist and leader in patient experience advancement, wrote in his 2016 piece, *Physician Burnout and Patient Experience: Flip Sides of the Same Coin*, “the answer to physician burnout is not to reduce aspirations for the care that we deliver to our patients… it is to become more ambitious.”^1^ Those who feel that they “care for all patients equally even when it is difficult” may not only be more resistant to burnout, but more resilient when facing stressful situations such as the COVID-19 pandemic.^2^ None of this needs to be time- or labor-intensive: the 40 seconds of compassion found by Johns Hopkins researchers to be enough to make a meaningful difference for patients is less time than it takes many of us to log into our medical record software.^3^,^4^

## GERIATRIC-SPECIFIC CHALLENGES

Not only is it widely accepted that geriatric patients bear a higher burden of morbidity and mortality with COVID-19, but older patients also face a greater number of challenges related to communication, compassion, and less-than-ideal care environments because of this pandemic.

While video and telephonic alternatives to bedside evaluation may facilitate communication with patients while maintaining physical separation to limit healthcare worker exposure and preserve PPE, these solutions may not be as effective in older patients, who commonly have hearing and visual impairment, challenges with manual dexterity due to arthritis, and cognitive impairment, all of which impede effective use of such technology. Accordingly, when caring for older adults, the healthcare provider may be faced with choosing between adequate communication and minimizing healthcare provider exposure and PPE usage.

Additionally, many healthcare facilities are enacting strict visitor restrictions to minimize the potential for nosocomial COVID-19 spread. While these decisions may be appropriate from an institutional level, they take a significant emotional toll on patients, family members, and the care providers tasked with communicating these policies. For patients with cognitive impairment, there is also substantial medical risk associated with being separated from family members or caregivers: those with dementia may demonstrate behaviors such as fidgeting, wandering, or increased aggression to communicate pain, hunger, or need for toileting, and without the presence of family at the bedside to aid in interpretation, these needs are at risk of going unmet.^5^–^8^ To alleviate this, some facilities do allow exemptions to current visitor restrictions, including for patients with dementia. For spouses of older patients, this may leave them with an impossible choice – to stay at the bedside to comfort their loved one and facilitate care, or to stay home and reduce their own risk of personal exposure.

Lastly, while pharmacologic treatment of agitation in patients with dementia or delirium is typically reserved for those with severe symptoms given the associated risk of death in older adults, more aggressive management with antipsychotic medications may become commonplace in the setting of COVID-19. The inability of staff to spend time – and valuable PPE – at the bedside to provide redirection, combined with the absence of those family members at the bedside who so often provide comfort and reassurance, may lead to pharmacologic intervention for less severe agitation than under usual conditions.^9^

## POTENTIAL INTERVENTIONS

Despite these challenges, emergency clinicians have at their disposal a myriad of techniques that have previously proven effective, and multiple opportunities for patient care optimization exist. In fact, many of the benefits of increased focus on communication, empathy, and compassion may be enhanced during the COVID-19 crisis, underscoring the need to focus effort and, as necessary, resources, on their expansion.

### Patient Arrival

While patients arriving to the ED are greeted by necessary, strongly worded signage and physical barriers reminding them of the need to maintain distancing and to immediately make staff aware of infectious symptoms, as well as reminding them of visitor exclusion policies, the communication techniques used by front-end staff to welcome patients are critical. Simple, scripted language for triage staff reminding patients that infection control is being emphasized to maintain their own and the staff’s safety, and that despite isolation precautions they will not be forgotten, may allow patients to adjust their expectations compared to usual circumstances, enhancing their satisfaction and reducing frustration throughout their visit.^10^,^11^

Signage communicating infection control policies is critical, and print must be large enough to be easily deciphered by geriatric patients, and medical interpreters must be readily available to provide information in non-English languages. These steps serve not only to improve understanding and reduce the risk of inadvertent policy lapses, but to improve overall engagement with care and patient experience. Multiple studies suggest that, taken together, these factors also improve patient outcomes.^12^–^15^

### Clinician Evaluation

Even as many EDs have moved rapidly toward using tablet-based video conferencing and other technology-based solutions to reduce time spent by clinicians and staff at the bedside, staff can – and should – still employ empathic communication techniques to comfort patients at times of maximal stress. The importance of each member of the care team introducing themselves, whether evaluating the patient in person or by video, cannot be overstressed. Given the anonymity caused by extensive PPE use, including face shields and masks, preexisting challenges with making ED patients aware of individual care-team roles are exacerbated.^16^

Reminder mnemonics such as ICARE (Introduction and stating role in care, Collaboration with patients, Acknowledgment of emotions and the situation, Reflective listening, and Expectation setting) or AIDET (Acknowledge, Introduce, Duration, Explanation, Thank you) may be particularly useful heuristic tools during the COVID-19 pandemic when capacity is already stretched thin.^17^,^18^ As nursing staff generally spend more time at the bedside than physicians and other providers and are subject to the same – or more significant – limitations in bandwidth, it is vital that they be included in any efforts to improve communication in current circumstances.^19^

Finally, to maximize the positive perceived empathy of the brief period spent at the bedside, sitting (eg, on a stool) as opposed to standing is imperative; this simple act increases patients’ perceptions of both the time spent with them by care providers and of clinician compassion.^20^,^21^

### Discharge Planning

While most patients evaluated for COVID-19 in the ED are medically appropriate for discharge home, symptomatic patients may suffer from fear and uncertainty about their expected course of illness. Using uniform written discharge instructions specifically for patients either confirmed to have COVID-19 or awaiting testing results may have many benefits.^22^ Not only will reducing variability improve adherence to isolation and other outpatient management recommendations, but providing frontline staff with comprehensive, pre-written instructions reduces the work burden associated with individual patient discharges and allows for the inclusion of extended information surrounding expected disease course, follow-up planning, and support resources for those suffering from the psychological effects of isolation or requiring local resources such as access to food. Again, provision of information in patients’ native languages is obligatory to ensure optimal comprehension.^23^

As many patients will be discharged despite ongoing symptoms and – as hospital capacity constraints grow – even potentially with evidence of moderate disease, post-discharge follow-up calls, in which a staff member contacts patients by phone following ED discharge, will continue to serve multiple purposes. First, identification of worsening symptoms such as shortness of breath or weakness may prompt recommendation to return to the ED for reevaluation. Second, engaging patients by phone following discharge serves to remind them of the compassionate care they received in the ED, potentially mitigating the negative psychological effects of ongoing home isolation.^24^

## CONCLUSION

The 2020 novel coronavirus pandemic presents an unprecedented challenge to emergency care clinicians and staff as well as to ED patients and their families. Given the significant fear, anxiety, and uncertainty that has accompanied the proliferation of the virus across the world, there is no more important time to focus on best practices surrounding communication, empathy, and compassion. This is particularly critical for geriatric patients who find themselves at highest risk of injury – both physiologic and psychologic – from this crisis. Fortunately, numerous opportunities exist to make small but meaningful practice changes with the potential of dramatically improving our patients’ and staff’s experience. While the fundamental disruptions to all of our lives brought by COVID-19 are hopefully temporary, the gains we make in maximizing humanism in our care can benefit our patients indefinitely.
